# A Novel Serum Metabolomic Profile for the Differential Diagnosis of Distal Cholangiocarcinoma and Pancreatic Ductal Adenocarcinoma

**DOI:** 10.3390/cancers12061433

**Published:** 2020-05-31

**Authors:** Rocio I.R. Macias, Luis Muñoz-Bellvís, Anabel Sánchez-Martín, Enara Arretxe, Ibon Martínez-Arranz, Ainhoa Lapitz, M. Laura Gutiérrez, Adelaida La Casta, Cristina Alonso, Luis M. González, Matias A. Avila, Maria L. Martinez-Chantar, Rui E. Castro, Luis Bujanda, Jesus M. Banales, Jose J.G. Marin

**Affiliations:** 1 Experimental Hepatology and Drug Targeting (HEVEFARM) Group, University of Salamanca, Biomedical Research Institute of Salamanca (IBSAL), 37007 Salamanca, Spain; anabelsanchez@usal.es (A.S.-M.); jjgmarin@usal.es (J.J.G.M.); 2 National Institute for the Study of Liver and Gastrointestinal Diseases (CIBERehd, Carlos III Health Institute), 28029 Madrid, Spain; maavila@unav.es (M.A.A.); mlmartinez@cicbiogune.es (M.L.M.-C.); luis.bujandafernandezdepierola@osakidetza.eus (L.B.); jesus.banales@biodonostia.org (J.M.B.); 3 Service of General and Gastrointestinal Surgery, University Hospital of Salamanca, IBSAL, CIBERONC, 37007 Salamanca, Spain; luismb@usal.es (L.M.-B.); lgfcir@gmail.com (L.M.G.); 4 OWL Metabolomics, Bizkaia Technology Park, 48160 Derio, Spain; earretxe@owlmetabolomics.com (E.A.); imartinez@owlmetabolomics.com (I.M.-A.); calonso@owlmetabolomics.com (C.A.); 5 Department of Liver and Gastrointestinal Diseases, Biodonostia Health Research Institute, Donostia University Hospital, University of the Basque Country (UPV/EHU), 20014 San Sebastian, Spain; ainhoa.lapitz@biodonostia.org (A.L.); ADELAIDA.LACASTAMUNOA@osakidetza.eus (A.L.C.); 6 Department of Medicine and Cytometry Service (NUCLEUS), University of Salamanca, Cancer Research Center (IBMCC-CSIC/USAL), IBSAL, CIBERONC, 37007 Salamanca, Spain; mlgutierrez@usal.es; 7 Program of Hepatology, Center for Applied Medical Research (CIMA), University of Navarra-IDISNA, 31008 Pamplona, Spain; 8 Liver Disease Lab Center for Cooperative Research in Biosciences (CIC bioGUNE), Basque Research and Technology Alliance (BRTA), Bizkaia Technology Park, 48160 Derio, Spain; 9 Research Institute for Medicines (iMed.ULisboa), Faculty of Pharmacy, Universidade de Lisboa, 1649-003 Lisbon, Portugal; ruieduardocastro@ff.ulisboa.pt; 10 IKERBASQUE, Basque Foundation for Science, 48013 Bilbao, Spain

**Keywords:** biliary cancer, differential diagnosis, metabolites, pancreatic cancer, tumor non-invasive biomarker

## Abstract

The diagnosis of adenocarcinomas located in the pancreas head, i.e., distal cholangiocarcinoma (dCCA) and pancreatic ductal adenocarcinoma (PDAC), constitutes a clinical challenge because they share many symptoms, are not easily distinguishable using imaging techniques and accurate biomarkers are not available. Searching for biomarkers with potential usefulness in the differential diagnosis of these tumors, we have determined serum metabolomic profiles in healthy controls and patients with dCCA, PDAC or benign pancreatic diseases (BPD). Ultra-high-performance liquid chromatography coupled to mass spectrometry (UHPLC-MS) analysis was performed in serum samples from dCCA (*n* = 34), PDAC (*n* = 38), BPD (*n* = 42) and control (*n* = 25) individuals, divided into discovery and validation cohorts. This approach permitted 484 metabolites to be determined, mainly lipids and amino acids. The analysis of the results led to the proposal of a logistic regression model able to discriminate patients with dCCA and PDAC (AUC value of 0.888) based on the combination of serum levels of nine metabolites (acylcarnitine AC(16:0), ceramide Cer(d18:1/24:0), phosphatidylcholines PC(20:0/0:0) and PC(O-16:0/20:3), lysophosphatidylcholines PC(20:0/0:0) and PC(0:0/20:0), lysophosphatidylethanolamine PE(P-18:2/0:0), and sphingomyelins SM(d18:2/22:0) and SM(d18:2/23:0)) and CA 19-9. In conclusion, we propose a novel specific panel of serum metabolites that can help in the differential diagnosis of dCCA and PDAC. Further validation of their clinical usefulness in prospective studies is required.

## 1. Introduction

Although distal cholangiocarcinoma (dCCA) and pancreatic ductal adenocarcinoma (PDAC) share a close anatomical location, they are considered distinct entities and require specific management strategies [1]. Whereas dCCA is an aggressive malignancy that arises in the biliary tract below the cystic duct and represents approximately 20% of CCAs, PDAC derives from the epithelium of pancreatic ducts and is the fourth cause of cancer-related deaths [2,3]. Although dCCA has a poor clinical outcome [4] due to its late diagnosis and resistance to chemotherapy [5], in general, the prognosis is worse in the case of PDAC [3].

Despite improvements in imaging techniques during recent years, the accurate diagnosis of adenocarcinomas located in the pancreas head area represents a clinical challenge in gastrointestinal oncology. Biopsy, either using cytologic brushing or fine-needle aspiration guided by endoscopic ultrasound, is mandatory to confirm the diagnosis. However, this has serious limitations: (i) repeat sampling is often required since the quality of the samples is not always sufficient to carry out the anatomopathological analysis, and (ii) the detection of malignant cells can confirm the diagnosis, but a negative result does not permit ruling it out [6]. To distinguish PDAC from benign pancreas diseases (BPD), such as chronic pancreatitis or pancreatic cysts, is also challenging, and the lack of accurate tumor biomarkers justifies that ≈ 5–10% of surgical removals of the head of the pancreas due to presumed malignancies are finally identified as benign lesions.

Several non-invasive biomarkers have been evaluated for the diagnosis of PDAC [7] and CCA [8,9], but none of them are being used in the clinical setting. Serum carbohydrate antigen 19-9 (CA 19-9) is the only FDA-approved biomarker for PDAC for both the follow-up of the therapeutic response [10] and for the detection of recurrence after surgery. Nevertheless, owing to its low sensitivity and specificity, CA 19-9 is far from being considered an optimal biomarker. Serum CA 19-9 is also used clinically to help in diagnosis and to monitor the response to therapy in biliary cancers, usually in combination with another unspecific marker, i.e., carcinoembryonic antigen (CEA). However, its accuracy is low and is not suitable for early detection. In addition, CA 19-9 can be elevated in patients with obstructive cholestasis, chronic liver and pancreatic diseases, and premalignant pancreatic lesions. Moreover, ≈ 10% of the Caucasian population with Lewis-negative phenotype do not express this biomarker [11].

Therefore, there is an urgent need to identify reliable minimally invasive biomarkers that can help in the differential diagnosis of dCCA and PDAC. An optimal biomarker would also be expected to contribute to the early detection of these cancers. The analysis of a large number of small metabolites in biological samples represents an interesting approach for identifying clinically relevant biomarkers for different diseases. In this context, the aim of the present study was to evaluate the usefulness of differences in serum metabolomic profiles between dCCA and PDAC, as well as between these severe malignancies and BPD and healthy individuals.

## 2. Results

### 2.1. Characteristics of the Study Population

The demographic and clinical features of individuals from both cohorts are shown in Table 1. The age was higher in patients with dCCA and PDAC than in patients with BPD and healthy individuals and only the latter group included a lower percentage of males. Most tumors included in the dCCA group were in early stage, while there was a similar distribution of tumors in early and advanced stage in the PDAC group. Regarding liver biochemical parameters (Table 1), a significant increase in ALT, GGT, alkaline phosphatase and total bilirubin was found in patients with dCCA and PDAC. Except for total bilirubin, these parameters were also found to be elevated in BPD, although the magnitude of changes was lower than that observed in patients with tumors. 

A significant increase in serum levels of CA 19-9 was found in both dCCA and PDAC, with a marked interindividual variability. Moreover, although CA 19-9 levels were also elevated in some patients with BPD, both with pancreatic cysts and with chronic pancreatitis, these were significantly lower than those found in patients with cancer.

**Figure 1:**
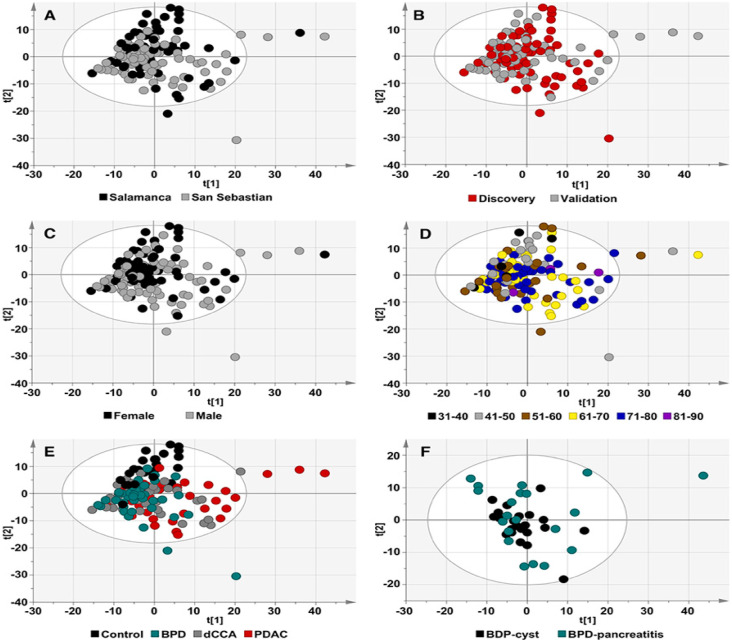
Principal component analysis (PCA) score plots of human serum samples. Colors represent (**A**) the origin of the samples, (**B**) discovery or validation cohort, (**C**) gender, (**D**) age range, (**E**) group of samples and (**F**) type of benign pancreatic disease (BPD). (**A**–**E**) Principal component 1 (t[1]) and principal component 2 (t[2]) explain 17.4% and 11.2% of the total variance, respectively. (**F**) t[1] and t[2] explain 19.8% and 13.1% of the total variance, respectively. Each dot represents one sample. The ellipse represents 95% confidence interval according to Hotelling’s T^2^ test.

Any clustering of the different groups of samples according to the serum metabolome was evaluated using multivariate data analysis, unsupervised principal component analysis (PCA) and supervised orthogonal partial least-squares to latent structures discriminant analysis (OPLS-DA) approaches. As shown in Figure 1, no differences in serum metabolomic profiles were found between the hospitals of origin, discovery and validation cohorts, gender, and group of age or group of samples (Figure 1A–E, respectively). A random distribution of patients with cysts and pancreatitis, both included in the BPD group, was found (Figure 1F). 

**Figure 2:**
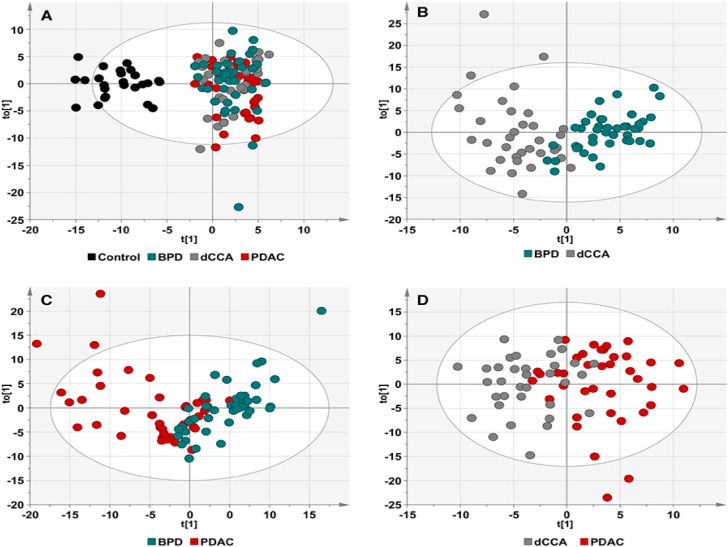
Score plots for the first predictive (t[1]) and orthogonal (to[1]) components of the supervised orthogonal partial least squares discriminant analysis (OPLS-DA) models for (**A**) disease vs. control samples; R^2^X = 0.445; R^2^Y = 0.835; Q^2^X = 0.694), (**B**) dCCA vs. BPD samples; R^2^X = 0.315; R^2^Y = 0.697; Q^2^X = 0.425, (**C**) PDAC vs. BPD samples; R^2^X = 0.265; R^2^Y = 0.471; Q^2^X = 0.163,and (**D**) dCCA vs. PDAC samples; R^2^X = 0.24; R^2^Y = 0.527; Q^2^X = 0.036. Each dot represents one sample. The ellipse represents 95% confidence interval according to Hotelling’s T^2^ test.

The supervised OPLS-DA model showed a good predictive ability to discriminate patient groups from healthy individuals, since Q^2^X = 0.694 (Figure 2A), triglycerides and, to a lesser extent, oxidized fatty acids and bile acids (all of them increased) and sphingomyelins and glycerophosphatidylcholines (both decreased) being the main contributors to the differences found between patients and control individuals. However, the supervised OPLS-DA models to differentiate dCCA vs. BPD patients, PDAC vs. BPD and both types of tumors showed very low predictive ability (Figure 2B–D, respectively), since Q^2^X values were low, especially in the comparisons of PDAC with BPD (Q^2^X = 0.163) and dCCA (Q^2^X close to 0).

### 2.2. Serum Metabolomic Profiles of Patients with dCCA, PDAC and BPD and Healthy Individuals

**Figure 3:**
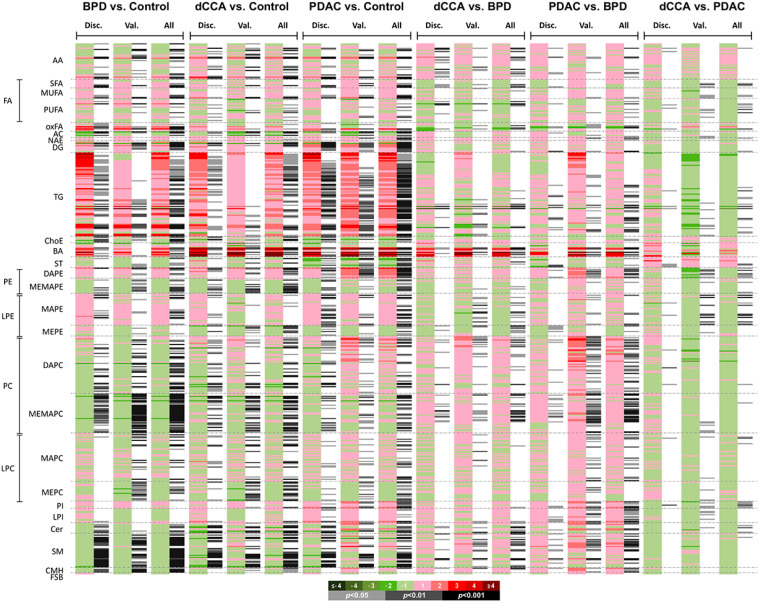
Metabolomic signatures in serum of patients with dCCA, PDAC or BPD and healthy individuals (Control). Heatmaps show fold-changes and *p*-values in each two-group comparison of the relative metabolite levels in serum samples in the discovery cohort (Disc.), in the validation cohort (Val.) and considering all samples together (All). The log_2_ transformed metabolite abundance ratios are depicted for each comparison. In the scale, colors from green to red correspond to drop or elevation of metabolite levels and gray lines show significant fold-changes of individual metabolites; darker gray colors indicate higher significance. Metabolites are grouped by chemical group/subgroup: AA, amino acids; AC, acylcarnitines; BA, bile acids; Cer, ceramides; ChoE, cholesteryl esters; CMH, monohexosylceramides; DAPC, diacylglycerophosphocholines; DAPE, diacylglycerophosphoethanolamines; DG, diglycerides; FSB, free sphingoid bases; LPC, lysophosphatidylcholines; LPE, lysophosphatidylethanolamines; LPI, lysophosphatidylinositols MAPC, monoacylglycerophosphatidylcholines; MAPE, monoacylglycerophosphatidylethanolamines; MEMAPC, 1-ether, 2-acylglycerophosphatidylcholines; MEMAPE, 1-ether, 2-acylglycerophosphatidylethanolamines; MEPC; 1-monoetherglycerophosphatidylcholines; MEPE, 1-monoetherglycerophosphatidylethanolamines; MUFA, monounsaturated fatty acids; NAE, N-acyl ethanolamines; oxFA, oxidized fatty acids; PC, phosphatidylcholines; PE, phosphatidylethanolamines; PI, phosphatidylinositols; PUFA, polyunsaturated fatty acids; SFA, saturated fatty acids; SM, sphingomyelins; ST, steroids; TG, triglycerides.

During the discovery phase we were able to determine 484 metabolites in serum samples, which was confirmed in the validation cohort. Changes in the levels of molecules belonging to the different families of analyzed metabolites (lipids, amino acids and amino acids derivatives) were found. Figure 3 depicts the heatmaps showing the fold-changes and the *p*-values generated from different two-groups comparisons carried out in the discovery and validation cohorts, and considering all samples together.

**Figure 4:**
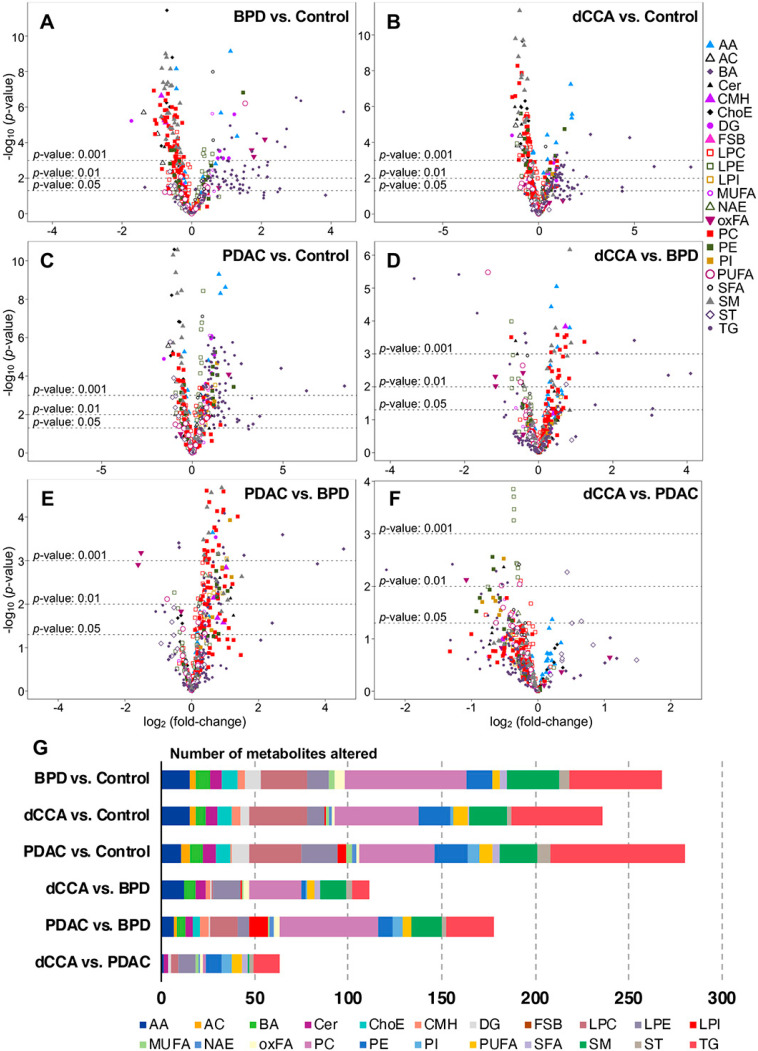
Volcano plots [-log_10_(*p*-value) and log_2_(fold-change)] considering the serum metabolite levels of the whole cohort of (**A**) BPD patients vs. controls, (**B**) dCCA vs. controls, (**C**) PDAC vs. controls, (**D**) dCCA vs. BPD, (**E**) PDAC vs. BPD and (**F**) dCCA vs. PDAC. (**G**) Number of metabolites and metabolite classes significantly different in each comparison. AA, amino acids; AC, acylcarnitines; BA, bile acids; Cer, ceramides; ChoE, cholesteryl esters; CMH, monohexosylceramides; DG, diglycerides; FSB, free sphingoid bases; LPC, lysophosphatidylcholines; LPE, lysophosphatidylethanolamines; LPI, lysophosphatidylinositols; MUFA, monounsaturated fatty acids; NAE, N-acyl ethanolamines; oxFA, oxidized fatty acids; PC, phosphatidylcholines; PE, phosphatidylethanolamines; PI, phosphatidylinositols; PUFA, polyunsaturated fatty acids; SFA, saturated fatty acids; SM, sphingomyelins; ST, steroids; TG, triglycerides.

Figure 4 shows the volcano plots generated for each two-groups comparison, and the number of metabolites significantly changed in each comparison considering the full cohort (Figure 4G). When BDL was compared with control, altered serum concentrations of 268 metabolites (mainly phosphatidylcholines > triglycerides > sphingomyelins ≈ lysophosphatidylcholines) were found. The comparison of dCCA with control revealed altered serum levels of 236 metabolites (mainly triglycerides ≈ phosphatidylcholines > lysophosphatidylcholines > sphingomyelins). 

The highest number of metabolites affected by changes in their serum levels (*n* = 280; mainly triglycerides > phosphatidylcholines > lysophosphatidylcholines) was found in the PDAC group. Different serum levels of 111 metabolites were found when comparing dCCA with BPD (mainly phosphatidylcholines > lysophosphatidyletanolamines = sphingomyelins), whereas this number increased to 178 when comparing PDAC with BPD (mainly phosphatidylcholines > triglycerides). The number of serum metabolites altered when comparing dCCA vs. PDAC was 63 (mainly triglycerides > phosphatidyletanolamines > lysophosphatidyletanolamines), and most of them were higher in PDCA than in dCCA. The number of metabolites with a value of area under the receiver operating characteristic curve (AUC) ≥ 0.8 was 73 when comparing BPD vs. control, 63 when comparing dCCA vs. control and 72 when comparing PDAC vs. control. 

An important number of metabolites were found altered in the serum of more than one group of patients, although the magnitude of changes was higher in patients with cancer. Table 2 shows the 10 metabolites with the best diagnostic capacity (best values of AUC, sensitivity and specificity) for each disease vs. control. Complete panels are presented in Table S1A–C. 

Although fewer alterations in the circulating metabolomic profiles were observed when the different diseases were cross compared, we found changes with interest in diagnosis. Among 50 metabolites with significant AUC values in the comparison of dCCA vs. BPD, 6 showed AUC values of ≥ 0.8 (Table 3), while 2 among 61 in the comparison PDAC vs. BPD reached these AUC values. In the comparison dCCA vs. PDAC, 9 metabolites showed significant AUC values, although all with AUC < 0.8. In the last comparison serum concentrations of the 9 metabolites were lower in dCCA than in PDAC. Table 3 shows the 9–10 metabolites with the best diagnostic capacity in each two-groups comparison, and the complete panels are presented in Table S1D,E.

### 2.3. Discrimination between Patients with and without Tumors

**Figure 5:**
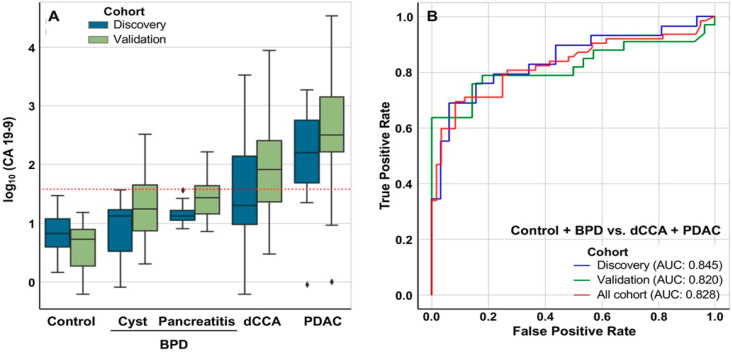
Diagnostic prediction capacity of CA 19-9 in tumors (dCCA+PDAC) vs. non tumors (Control+BPD). (**A**) Box plot diagrams show the log_10_ CA 19-9 (cut-off of 37 IU/mL). (**B**) Area under the receiver operating characteristic curve (AUC) in discovery and validation cohorts and considering all cohorts.

In our study, with a cut-off fixed in 37 IU/mL, CA 19-9 showed a good diagnostic capacity to differentiate patients with tumors (dCCA+PDAC) from healthy individuals, with an AUC of 0.93 in both cohorts. However, as shown in Figure 5A, it was not so good in differentiating between dCCA+PDAC and patients without cancer (Control+BPD). AUC was 0.845, 0.820 and 0.828 in discovery, validation and the whole cohort, respectively (Figure 5B).

**Figure 6:**
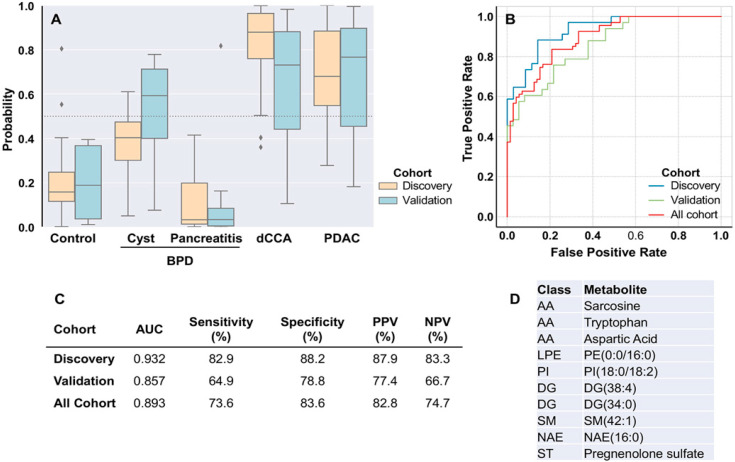
Diagnostic prediction capacity of the logistic model in tumors (dCCA+PDAC) vs. non tumors (Control+BPD). (**A**) Box plot diagrams showing the probability to detect each group as tumors. (**B**) Area under the receiver operating characteristic curve (AUC) in discovery and validation cohorts and considering all cohorts. (**C**) AUC, sensitivity, specificity, positive predictive value (PPV) and negative predictive value (NPV) of the algorithm to differentiate tumors vs. non tumors in each cohort. (**D**) Selected metabolites included in the model. AA, amino acids; DG, diglycerides; LPE, lysophosphatidylethanolamines; NAE, N-acyl ethanolamines; PI, phosphatidylinositols; SM, sphingomyelins; ST, steroids.

As shown in Figure 6, a model including 10 metabolites [amino acids sarcosine, tryptophan and aspartic acid, lysophosphatidylethanolamine PE(0:0/16:0), phosphatidylinositol PI(18:0/18:2), diglycerides DG(38:4) and DG(34:0), sphingomyelin SM(42:1), N-acyl ethanolamine NA(16:0) and sterol pregnenolone sulfate] was generated to differentiate patients with tumors (dCCA+PDAC) and without malignancies (Control+BPD). 

Using this model, the probability of diagnosing patients with chronic pancreatitis or healthy subjects as individuals suffering from dCCA or PDAC is low. However, this risk is higher for patients with benign pancreatic cysts (Figure 6A). AUC was 0.93 in discovery, 0.86 in validation and 0.89 considering the whole cohort. Sensitivity was 73.6% and specificity 83.6% considering the whole cohort. We have evaluated the relationship between the age and the diagnostic error rate of the model. Based on a stratification of the patients in quantiles, the diagnostic error rate was constant and around 20% (average 21%, ranging from the 17% to 29%) and was not associated with the patient’s age.

In our study, CA 19-9 showed a sensitivity of 71% and a specificity of 83% to differentiate patients with tumors from individuals without tumors (Controls+BPD).

### 2.4. Discrimination between dCCA and PDAC

Since none of the individual circulating metabolites had a sufficient capability of distinguishing dCCA from PDAC (Table 3), our next goal was to obtain a predictive model for discriminating between both tumors. A logistic regression model was built with nine metabolites (Figure S1) (acylcarnitine AC(16:0), ceramide Cer(d18:1/24:0), phosphatidylcholines PC(20:0/0:0) and PC(O-16:0/20.3), lysophosphatidylcholines PC(20:0/0:0) and PC(0:0/20:0), lysophosphatidylethanolamine PE(P-18:2/0:0), and sphingomyelins SM(d18:2/22:0) and SM(d18:2/23:0)), with an AUC of 0.91 in discovery, 0.82 in validation and 0.85 considering the whole cohort; sensitivity was 55.9% and specificity 89.5% considering all the patients. The analysis of CA 19-9 showed a sensitivity of 77% and a specificity of 48% to differentiate patients with PDAC from those with dCCA (Figure S2).

**Figure 7:**
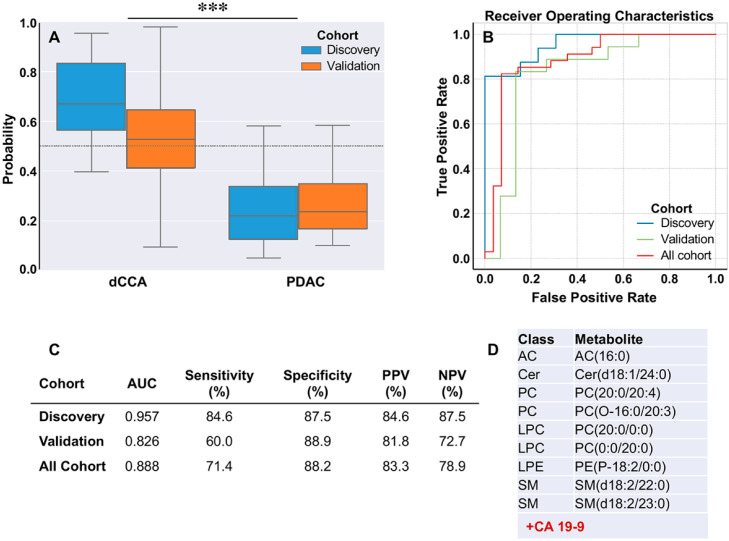
Diagnostic prediction capacity of the logistic model in dCCA vs. PDAC. (**A**) Box plot diagrams showing the probability to detect each type of tumor. (**B**) Area under the receiver operating characteristic curve (AUC) in discovery and validation cohorts and considering the whole cohort. (**C**) AUC, sensitivity, specificity, positive predictive value (PPV) and negative predictive value (NPV) of the algorithm to differentiate dCCA vs. PDAC in each cohort. (**D**) Selected metabolites included in the model: AC, acylcarnitine; Cer, ceramide; LPC, lysophosphatidylcholines; LPE, lysophosphatidylethanolamines; PC, phosphatidylcholines; SM, sphingomyelins plus CA 19-9. ***, *p* < 0.001.

Another logistic regression model was built with the nine metabolites plus CA 19-9 (Figure 7), which improved the sensitivity. However, the specificity slightly decreased in the full cohort and especially in the validation cohort. Thus, AUC was 0.888, sensitivity 71.4% and specificity 89.2 considering the whole cohort.

## 3. Discussion

The lack of non-invasive biomarkers for the early diagnosis of PDAC and dCCA contributes to the bad prognosis of these tumors [12]. The anatomical difficulty in accessing the tumors to obtain good quality biopsies for diagnostic purposes makes it necessary to identify minimally invasive biomarkers that could help, not only in the early detection of these tumors to enable more patients to benefit from surgical treatment, but also in the prognosis and follow-up of these patients during treatment. However, although important efforts have been made during recent years, none of the identified markers have been validated and reached clinical practice. Despite their moderate clinical utility, only CA 19-9 and carcinoembryonic antigen (CEA) are currently used for PDAC and CCA diagnosis [13].

Omics technologies are providing valuable information to understand cancer biology. Metabolic reprogramming is one hallmark of tumor cells [14,15]; thus, the analysis of the metabolome (hundreds of small molecules or metabolites) in body fluids of patients with cancer can give an indirect reflection of the metabolic behavior of the tumors and could be used to identify potential biomarkers. 

Several studies have been conducted to identify serum metabolomic profiles for the diagnosis of pancreatic or biliary cancers. Most of them included only patients with pancreatic cancer and healthy controls [16,17,18] or with biliary cancer and healthy individuals [19]. However, it is important to include clinically relevant controls since the metabolome can be affected by many factors, including gender, age, comorbidities, medication, life style, environment or circadian rhythms; in fact, important intra-day variations have been observed in serum levels of patients with advanced pancreatic cancer, which were further affected by cachexia [20].

The use of metabolomics to discriminate between different types of tumors and between tumors and benign diseases has been less explored. Combinations of metabolites discriminating malignant from benign pancreaticobiliary diseases and from healthy controls have been reported, although the number of cases was low and most of the patients with tumors were in an advanced stage, for which their usefulness in early diagnosis cannot be guaranteed [21]. More recently, a biomarker signature for the differential diagnosis between PDAC and chronic pancreatitis was reported, consisting of nine metabolites, five of them lipids (two sphingomyelins, sphinganine 1-phosphate, one phosphatidylcholine and one ceramide), and proline, histidine, pyruvate and isocitrate plus CA 19-9, with a negative predictive value of 99.9% in patients with chronic pancreatitis [22].

All these studies support the concept that the combination of several metabolite markers allows for a more accurate diagnosis. In this study, we have included patients with biopsy-proven tumors or cysts located in the head of the pancreas divided into two independent cohorts of PDAC, dCCA, BPD and controls. Although serum bile acids levels represented the most marked alteration in patients with cancer, this hypercholanemic condition occurs in different pathologies that are accompanied by cholestasis, in which compensatory mechanisms are developed to limit the accumulation and toxic effects of these compounds [23]. It has been demonstrated that obstructive jaundice impacts the performance of biomarkers for PDAC [24], and in our study, a certain degree of cholestasis was found in some patients with tumors, since serum bilirubin was elevated, and as a consequence, none of the bile acid species measured could be considered as a good biomarker.

In the present study we have identified a multimarker signature for the differential diagnosis of adenocarcinomas located in the pancreas including nine metabolites plus CA 19-9 with better performance than serum CA 19-9 alone and another panel of ten metabolites (seven lipids and three amino acids) with similar performance to serum CA 19-9 to discriminate tumors from BPD but which are especially useful for chronic pancreatitis. Since this disease is a risk factor for the development of pancreatic cancer [25], these biomarkers could be useful for early detection of tumor development, for monitoring patients during treatment and for avoiding unnecessary pancreatic surgery and its complications. Interestingly, some of the metabolites included in the signature proposed here belonged to the same families of compounds (amino acids, sphingomyelins and ceramides) of a previously described model [22]. Changes in serum levels of certain amino acids have been described in other tumors, such as liver [26,27] and breast [28] cancer. In addition, sphingomyelins and ceramides have been found altered in the serum of patients with liver [27] and ovarian [29] cancer. Alterations in sphingolipid metabolism have been associated with cell proliferation [30]. Our model of changes in ten metabolites was less accurate than CA 19-9 levels in distinguishing pancreatic cysts from tumors in the head of pancreas, although the low number of cases of cystic lesions in our cohort can be considered a limitation. In recent years, several studies have proposed circulating microRNA (miRNA) signatures for early detection of pancreatic cancer [31] or for the differential diagnosis of PDAC and chronic pancreatitis with good sensitivity and specificity [32], although none of them included a group of patients with pancreatic cysts. A recent study proposed a two-miRNA panel of downregulated miR-16 and upregulated miR-877 to differentiate patients with dCCA from benign disease (AUC = 0.90) and from PDAC (AUC = 0.88) [33]. Serum proteins have also been investigated. The analysis of cell migration-inducing hyaluronan binding protein (CEMIP) plus CA 19-9 improved the diagnostic value compared to CA 19-9 alone for the diagnosis of pancreatic cancer [34]; the study included a small but very heterogeneous group of patients with BPD in the control cohort, but the results must be validated.

In sum, in this study, using two independent cohorts of patients, we have identified a model consisting of 9 metabolites in serum with promising capability to differentiate both types of pancreatic head adenocarcinomas, with AUC = 0.854. Because accurate diagnosis of these tumors remains challenging, our results suggest that the analysis of multiple types of biomarkers could help in the early and differential diagnosis and in the follow-up of these aggressive tumors.

## 4. Materials and Methods

### 4.1. Study Population and Eligibility

Fasting serum samples from dCCA (*n* = 34), PDAC (*n* = 38), BPD (*n* = 42), and healthy subjects (*n* = 25) were obtained from two Spanish hospitals; University Hospital of Salamanca, National DNA Bank Carlos III, and Donostia University Hospital in San Sebastian. Samples were randomly divided in two cohorts, “discovery” and “validation”, with equal proportional representation of individuals belonging to each pathology as well as to each origin of samples. 

Inclusion criteria for patients with dCCA and PDAC were histopathologic confirmation of diagnosis by expert pathologists and serum obtained before any type of treatment. Exclusion criteria were other types of CCA or synchronous presence of another type of malignancy. The BPD group included 22 samples from patients with cysts and 20 from patients with chronic pancreatitis. Selected healthy individuals had no history of any type of malignancy and no clinical evidence of hepatopancreaticobiliary disease. Clinical and laboratory test values were collected from the patients’ records. The research protocol was approved by the Ethics Committee for Clinical Research of Salamanca (July 18, 2018) and San Sebastian (October 16, 2019), and informed written consent for the samples to be used for biomedical research was obtained from each patient.

### 4.2. Metabolomic Analyses 

Serum metabolic profiles were analyzed as previously described [35]. Briefly, two ultrahigh-performance liquid chromatography (UHPLC)-time of flight-MS based platforms analyzing methanol and chloroform/methanol serum extracts were combined with the amino acid measurement using an UHPLC-single quadrupole-MS based analysis. Identified ion features in the methanol extract platform included amino acids and its derivatives and lipids.

Metabolite extraction procedures, chromatographic separation conditions and mass spectrometric detection conditions have been previously described [35]. Metabolomics data were pre-processed using the TargetLynx application manager for MassLynx 4.1 (Waters Corp., Milford, MA, USA). Intra- and inter-batch normalization was performed by inclusion of multiple internal standards and pool calibration response correction, following a previously described procedure [36]. Data quality was assessed by the inclusion of quality control samples, including repeated injections of these samples to evaluate the reproducibility of the analysis process [36].

### 4.3. Statistical Analysis

Data are shown as mean ± SD. Differences between groups were determined using the Student´s *t*-test or the Bonferroni method of multiple range test, as appropriate. Calculations were performed using the statistical software package R v.3.4.0 (R Development Core Team, 2017; http://cran.r-project.org).

Multivariate principal component analysis (PCA) [37] and orthogonal partial least squares discriminant analysis (OPLS-DA) [38] modeling were performed with the software SIMCA 14.1 (Umetrics, Malmo, Sweden). Model quality was assessed using R^2^ and Q^2^ values, which indicate the explained fraction of variance and the goodness of prediction, respectively. The Q^2^ parameter was calculated by sevenfold cross validation.

To find statistical models to differentiate patients with tumors (dCCA or PDAC) and subjects without tumors (controls or BPD [chronic pancreatitis or pancreatic cysts]), as well as to differentiate each type of tumor, dCCA vs. PDAC, generalized linear models (GLM) were used and those selected were confirmed by leave-one-out cross validation (LOOCV). Box-Cox transformations were applied to the biomarker metabolite levels for correcting non-normally distributed data and used to calculate the classification algorithm. The diagnostic accuracy of the model to identify patients in each comparison was assessed using the AUC *p* < 0.05.

## 5. Conclusions

Based on the results obtained in the present study, we propose novel specific panels of serum metabolites that can help in the early and differential diagnosis of dCCA and PDAC. Further validation of their clinical usefulness in prospective studies including other relevant controls and in combination with clinical features is required.

## Figures and Tables

**Table 1:** Demographic and clinical characteristics of the discovery and validation cohorts.

Variable	Control		BPD		dCCA		PDAC	
	Discovery (*n* = 12)	Validation (*n* = 13)	Discovery (*n* = 22)	Validation (*n* = 20)	Discovery (*n* = 16)	Validation (*n* = 18)	Discovery (*n* = 19)	Validation (*n* = 19)
Age, mean ± SD	53.7 ± 9.8	52.2 ± 8.6	60.9 ± 12.3	59.9 ± 11.6	70.9 ± 7.0	68.6 ± 10.9	68.5 ± 9.3	63.1 ± 9.4
Males, *n* (%)	5 (41.7)	6 (46.1)	12 (54.5)	10 (50)	12 (75)	10 (55.6)	12 (63.1)	12 (63.1)
**Tumor stage** *, *n* (%)								
I	-	-	-	-	0 (0)	1 (5.6)	1 (5.3)	2 (10.5)
II	-	-	-	-	13 (81.2)	16 (88.8)	7 (36.8)	7 (36.8)
III	-	-	-	-	1 (6.3)	0 (0)	2 (10.5)	5 (26.3)
IV	-	-	-	-	2 (12.5)	1 (5.6)	9 (47.4)	5 (26.3)
**Biochemistry**, mean ± SD								
ALT (IU/L)	21.3 ± 8.0	17.1 ± 6.7	38.9 ± 64.3	32.0 ± 27.0	107 ± 118 ^a,b^	38.5 ± 94.5	207 ± 203 ^a,b^	208 ± 318 ^a,b^
GGT (IU/L)	24.8 ± 20.4	19.0 ± 9.8	125 ± 206 ^a^	100 ± 142 ^a^	589 ± 592 ^a,b^	411 ± 816 ^a,b^	775 ± 1037 ^a,b^	784 ± 978 ^a,b^
Alkaline phosphatase (IU/L)	56.1.8 ± 15.4	58.5 ± 22.9	102 ± 115	94 ± 68	301 ± 220 ^a,b^	207 ± 153 ^a^	442 ± 427 ^a,b^	380 ± 359 ^a,b^
Total bilirubin (mg/dL)	0.5 ± 0.3	0.6 ± 0.2	0.5 ± 0.2	0.8 ± 1.7	6.9 ± 7.5 ^a,b^	2.8 ± 4.9 ^a^	7.0 ± 7.6 ^a,b^	7.3 ± 6.2, ^a,b^
CA 19-9 (IU/mL)	5.4 ± 4.5	8.6 ±7.4	46.6 ± 74.9 ^a^	15.1 ± 10.5	893 ± 2405 ^a,b^	328 ± 855 ^a,b^	2983 ± 8024 ^a,b^	431 ± 561 ^a,b^

^a^, *p* < 0.05 compared with control (in the same cohort) and ^b^, *p* < 0.05 compared with BPD (in the same cohort) using the Bonferroni method of multiple range test. *, AJCC Cancer Staging Manual, 7th Edition. ALT; alanine aminotransferase, BPD, benign pancreatic disease; CA 19-9, carbohydrate antigen 19-9; dCCA, distal cholangiocarcinoma; GGT, gamma-glutamyl transpeptidase; PDAC, pancreatic ductal adenocarcinoma.

**Table 2:** Diagnostic capacity of the top 10 metabolites in the comparison of each disease vs. control considering the whole cohort.

BPD vs. Control	**Metabolite**	**AUC**	**Sensitivity**	**Specificity**	**log_2_FC**
	Glutamic acid	0.926	90	84	1.112
	Tryptophan	0.910	92	83	−0.441
	DG(34:0)	0.910	84	86	−1.731
	PE(16:0/18:1)	0.909	88	88	1.475
	SM(32:1)	0.909	96	74	−0.722
	AC(8:0)	0.906	92	86	−1.379
	PC(O-16:0/18:2)	0.903	92	71	−1.087
	Arachidic acid	0.898	69	96	0.606
	SM(d18:2/22:0)	0.896	84	83	−0.749
	SM(38:1)	0.889	96	76	−0.612
dCCA vs. Control	**Metabolite**	**AUC**	**Sensitivity**	**Specificity**	**log_2_FC**
	SM(d18:2/22:0)	0.967	92	94	−0.992
	SM(d18:2/23:0)	0.959	88	97	−1.204
	SM(39:1)	0.958	96	91	−1.052
	Aspartic acid	0.955	79	100	1.671
	Glycocholic acid	0.954	94	88	4.779
	SM(38:1)	0.951	96	94	−0.750
	SM(d18:1/23:0)	0.929	80	94	−0.900
	SM(d18:1/22:0)	0.928	92	88	−0.810
	SM(d18:2/20:0)	0.921	84	88	−0.581
	Taurocholic acid	0.919	76	100	8.035
PDAC vs. Control	**Metabolite**	**AUC**	**Sensitivity**	**Specificity**	**log_2_FC**
	Glutamic acid	0.937	92	88	1.570
	Aspartic acid	0.937	79	96	1.473
	PE(16:0/18:1)	0.919	82	88	2.295
	SM(d18:2/22:0)	0.919	92	89	−0.810
	SM(39:1)	0.915	88	82	−0.907
	SM(d18:2/23:0)	0.911	92	87	−1.059
	ChoE(18:3)	0.907	76	92	−1.178
	AC(8:0)	0.903	80	92	−1.312
	SM(38:1)	0.899	96	76	−0.603
	PE(16:0/0:0)	0.897	79	92	0.601

AUC, area under the receiver operating characteristic curve; FC, fold change. Colors from green to red correspond to drop or elevation of metabolite levels.

**Table 3:** Diagnostic capacity of the top 9-10 metabolites in each two-disease group comparison considering the whole cohort.

dCCA vs. BPD	**Met** **a** **bolite**	**AUC**	**Sensitivity**	**Specificity**	**log_2_FC**
	SM(d18:1/23:1)	0.858	79	81	0.839
	Glycocholic acid	0.834	94	62	2.579
	Taurocholic acid	0.823	73	83	4.096
	PC(16:0/16:0)	0.811	76	79	0.753
	PC(31:0)	0.805	71	81	1.233
	TG(54:7)	0.800	60	91	−3.358
	18:3n-3	0.790	69	82	−1.368
	CMH(d18:1/16:0)	0.788	82	71	0.720
	Phenylalanine	0.785	56	90	0.482
	TG(54:6)	0.783	51	100	−2.154
PDAC vs. BPD	**Met** **a** **bolite**	**AUC**	**Sensitivity**	**Specificity**	**log_2_FC**
	PC(O-34:1)	0.814	66	90	0.951
	SM(d18:1/23:1)	0.813	79	79	0.898
	PC(P-16:0/16:0)	0.795	74	74	0.730
	PC(16:0/16:0)	0.794	66	86	0.950
	PC(31:0)	0.794	68	81	1.376
	PC(O-16:0/16:0)	0.782	68	81	0.941
	PC(O-38:5)	0.782	58	93	0.446
	PC(O-18:1/18:1)	0.776	63	81	0.760
	PC(O-22:1/20:4)	0.768	74	71	0.677
	SM(d18:0/15:0)	0.766	55	93	1.058
dCCA vs. PDAC	**Met** **a** **bolite**	**AUC**	**Sensitivity**	**Specificity**	**log_2_FC**
	PE(18:0/0:0)	0.769	82	71	−0.367
	PE(0:0/18:0)	0.763	84	68	−0.358
	PE(16:0/0:0)	0.742	74	68	−0.356
	PE(20:4/0:0)	0.739	74	71	−0.317
	PE(0:0/20:4)	0.735	76	71	−0.302
	PE(0:0/16:0)	0.732	89	47	−0.365
	PE(38:5)	0.719	95	44	−0.844
	TG(52:5)	0.705	71	68	−0.620
	PE 20:4	0.704	95	38	−0.520

AUC, area under the receiver operating characteristic curve; FC, fold change. Colors from green to red correspond to drop or elevation of metabolite levels.

## Supplementary Materials

The following are available online at https://www.mdpi.com/2072-6694/12/6/1433/s1, Figure S1: Diagnostic prediction capacity of the model of nine metabolites in dCCA vs. PDAC, Figure S2: Diagnostic prediction capacity of CA 19-9 in dCCA vs. PDAC, Table S1: Diagnostic capacity of the metabolites in the comparison.

## Abbreviations

AUC	area under the receiver operating characteristic curve
BPD	benign pancreatic disease
CA 19-9	carbohydrate antigen 19-9
CCA	cholangiocarcinoma
dCCA	distal cholangiocarcinoma
OPLS	orthogonal partial least squares
PCA	principal component analysis
PDAC	pancreatic ductal adenocarcinoma
UHPLC-MS	ultra-high performance liquid chromatography coupled to mass spectrometry
