# Supplementary material for: A Novel Serum Metabolomic Profile for the Differential Diagnosis of Distal Cholangiocarcinoma and Pancreatic Ductal Adenocarcinoma
Source: Cancers (Basel). 2020 May 31;12(6):1433. doi: 10.3390/cancers12061433 (PMC7352809; doi:10.3390/cancers12061433)
Supplement: Supplementary file 1 [file cancers-12-01433-s001.pdf]

Supplementary Materials

A Novel Serum Metabolomic Profile for the Differential Diagnosis of Distal Cholangiocarcinoma and Pancreatic Ductal Adenocarcinoma

Rocio I.R. Macias, Luis Muñoz-Bellvís, Anabel Sánchez-Martín, Enara Arretxe, Ibon Martínez-Arranz, Ainhoa Lapitz, M. Laura Gutiérrez, Adelaida La Casta, Cristina Alonso, Luis M. González, Matias A. Avila, Maria L. Martinez-Chantar, Rui E. Castro, Luis Bujanda, Jesus M. Banales, Jose J.G. Marin

Table S1A. Diagnostic capacity of the metabolites in the comparison of BPD vs. Control considering the whole cohort.

Class	Metbolite	AUC	Sensitivity	Specificity	log_2FC
Amino acids	Glutamic Acid	0.926	90	84	1.112
Amino acids	Tryptophan	0.910	92	83	−0.441
Glycerolipids	DG(34:0)	0.910	84	86	−1.731
Glycerophospholipids	PE(16:0/18:1)	0.909	88	88	1.475
Sphingolipids	SM(32:1)	0.909	96	74	−0.722
Fatty esters	AC(8:0)	0.906	92	86	−1.379
Glycerophospholipids	PC(O-16:0/18:2)	0.903	92	71	−1.087
Fatty acids	Arachidic acid	0.898	69	96	0.606
Sphingolipids	SM(d18:2/22:0)	0.896	84	83	−0.749
Sphingolipids	SM(38:1)	0.889	96	76	−0.612
Sphingolipids	SM(39:1)	0.886	72	90	−0.858
Glycerolipids	TG(58:3)	0.884	79	84	2.721
Sphingolipids	CMH(d18:1/22:0)	0.881	76	90	−0.872
Glycerophospholipids	PC(40:8)	0.878	84	79	−0.906
Glycerolipids	TG(58:2)	0.876	74	88	2.680
Glycerophospholipids	PC(O-16:0/20:4)	0.874	80	83	−0.720
Sphingolipids	SM(d18:1/22:0)	0.874	88	79	−0.610
Glycerophospholipids	PC(O-16:0/14:0)	0.871	76	88	−0.772
Glycerolipids	TG(56:2)	0.870	95	68	1.867
Glycerophospholipids	PC(P-16:0/18:2)	0.868	88	76	−0.684
Sphingolipids	SM(d18:1/23:0)	0.865	80	81	−0.727
Sterols	ChoE(16:0)	0.864	92	74	−0.470
Sphingolipids	SM(33:1)	0.862	96	62	−0.688
Sphingolipids	SM(d18:2/16:0)	0.861	80	81	−0.570
Fatty esters	AC(12:0)	0.861	96	71	−0.982
Glycerolipids	DG(34:2)	0.860	81	80	1.222
Amino acids	Tryptophan	0.858	84	79	−0.439
Sphingolipids	SM(d18:2/23:0)	0.858	88	71	−0.820
Sterols	ChoE(18:3)	0.857	76	86	−0.871
Glycerophospholipids	PC(O-38:5)	0.857	64	100	−0.566
Glycerolipids	TG(58:4)	0.857	74	92	3.132
Glycerophospholipids	PC(O-42:6)	0.853	92	64	−1.011
Sphingolipids	SM(42:1)	0.850	80	83	−0.628
Sphingolipids	CMH(d18:1/24:0)	0.849	88	79	−0.743
Sphingolipids	SM(31:1)	0.848	60	98	−0.782
Glycerophospholipids	PC(P-16:0/16:0)	0.845	92	67	−0.458
Glycerophospholipids	PC(O-18:0/18:2)	0.845	64	95	−1.053
Glycerolipids	TG(60:3)	0.845	79	88	2.454
Sphingolipids	CMH(d18:1/23:0)	0.844	80	79	−0.776
Glycerophospholipids	PC(O-18:1/18:2)	0.842	68	90	−0.838

Glycerophospholipids	PC(O-40:5)	0.842	84	74	−0.702
Fatty acids	24:1n-9	0.842	71	88	0.591
Glycerophospholipids	PC(O-22:0/20:4)	0.841	64	95	−0.792
Glycerophospholipids	PC(18:2/20:4)	0.840	76	79	−0.752
Glycerophospholipids	PC(O-22:1/20:4)	0.840	64	90	−0.752
Glycerolipids	TG(56:1)	0.840	69	88	2.184
Glycerophospholipids	PC(O-20:0/20:4)	0.838	64	90	−0.757
Sphingolipids	SM(d18:1/16:0)	0.838	84	74	−0.337
Glycerophospholipids	PC(18:2/18:2)	0.837	64	98	−1.053
Glycerolipids	TG(60:2)	0.832	79	80	2.168
Glycerophospholipids	PC(O-18:1/22:4)	0.830	80	76	−0.472
Sphingolipids	SM(d18:1/12:0)	0.828	80	79	−0.706
Amino acids	Aspartic Acid	0.827	90	68	0.837
Sphingolipids	SM(d18:2/20:0)	0.827	76	83	−0.504
Glycerophospholipids	PC(O-18:0/20:4)	0.825	76	81	−0.666
Oxidized fatty acids	12-HETE	0.825	62	96	2.098
Glycerolipids	TG(54:6)	0.824	73	88	2.998
Sterols	ChoE(22:4)	0.822	84	76	−0.732
Sphingolipids	SM(d18:2/14:0)	0.822	84	76	−0.649
Glycerolipids	TG(58:1)	0.822	69	84	1.914
Sphingolipids	SM(d18:0/15:0)	0.821	72	90	−0.739
Sphingolipids	SM(43:1)	0.818	76	79	−0.873
Sphingolipids	SM(d18:1/17:0)	0.816	84	76	−0.489
Sphingolipids	SM(d18:1/18:0)	0.815	80	76	−0.404
Fatty acids	18:3n-3	0.812	74	92	1.533
Glycerophospholipids	PC(15:0/20:4)	0.810	60	90	−0.808
Glycerophospholipids	PC(20:0/20:4)	0.809	88	64	−0.488
Fatty esters		0.809	80	81	−0.832
Glycerophospholipids	PC(O-24:1/20:4)	0.807	64	90	−0.698
Glycerophospholipids	PC(P-16:0/14:0)	0.805	72	81	−0.591
Glycerophospholipids	PC(P-36:2)	0.802	88	62	−0.595
Glycerolipids	TG(54:7)	0.801	62	100	4.358
Glycerophospholipids	PC(P-16:0/20:4)	0.800	72	86	−0.444

AUC, area under the receiver operating characteristic curve; FC, fold change. Colors from green to red correspond to drop or elevation of metabolite levels.

Table S1B. Diagnostic capacity of the metabolites in the comparison of dCCA vs. Control considering the whole cohort.

Class	Metabolite	AUC	Sensitivity	Specificity	log_2FC
Sphingolipids	SM(d18:2/22:0)	0.967	92	94	−0.992
Sphingolipids	SM(d18:2/23:0)	0.959	88	97	−1.204
Sphingolipids	SM(39:1)	0.958	96	91	−1.052
Amino acids	Aspartic Acid	0.955	79	100	1.671
Bile acids	Glycocholic acid	0.954	94	88	4.779
Sphingolipids	SM(38:1)	0.951	96	94	−0.750
Sphingolipids	SM(d18:1/23:0)	0.929	80	94	−0.900
Sphingolipids	SM(d18:1/22:0)	0.928	92	88	−0.810
Sphingolipids	SM(d18:2/20:0)	0.921	84	88	−0.581
Bile acids	Taurocholic acid	0.919	76	100	8.035
Glycerophospholipids	PC(O-18:0/18:2)	0.916	96	71	−1.361
Amino acids	Glutamic Acid	0.914	76	100	1.739
Sphingolipids	SM(42:1)	0.911	92	88	−0.873
Sphingolipids	Cer(d18:1/24:0)	0.905	100	71	−1.109
Fatty esters	AC(8:0)	0.904	92	82	−1.183
Glycerophospholipids	PC(20:3/20:4)	0.898	100	65	−1.211
Glycerophospholipids	PC(40:8)	0.898	84	85	−1.104
Sphingolipids	Cer(43:1)	0.898	80	85	−1.232
Glycerophospholipids	PC(O-16:0/20:3)	0.895	72	97	−0.885
Bile acids	GCDCA	0.893	74	96	2.751
Bile acids	TCDCA	0.887	79	88	6.126
Sphingolipids	SM(43:1)	0.881	76	85	−1.043
Glycerophospholipids	PC(20:0/0:0)	0.875	88	79	−0.608
Glycerolipids	DG(34:0)	0.869	80	85	−1.364
Glycerophospholipids	PC(O-16:0/18:2)	0.866	72	91	−1.026
Sphingolipids	Cer(d18:1/25:0)	0.861	72	91	−0.918
Glycerophospholipids	PE(O-16:0/0:0)	0.859	92	74	−1.107
Sphingolipids	Cer(d18:1/23:0)	0.859	80	82	−0.877
Fatty acids	24:1n-9	0.855	85	84	0.826
Glycerophospholipids	PC(0:0/20:0)	0.854	84	79	−0.551
Sphingolipids	SM(32:1)	0.853	80	85	−0.605
Sphingolipids	SM(d18:2/14:0)	0.852	84	82	−0.727
Peptides	Gly-Gly	0.851	68	96	1.756
Sphingolipids	SM(d18:1/25:0)	0.848	80	88	−0.707
Bile acids	TUDCA + THDCA	0.846	68	100	4.344
Fatty esters	AC(12:0)	0.845	76	79	−0.945
Sterols	ChoE(18:3)	0.844	92	65	−0.928
Glycerophospholipids	PC(P-17:0/20:4)	0.842	72	94	−0.673
Glycerophospholipids	PE(P-18:0/20:4)	0.841	72	94	−0.922
Glycerophospholipids	PE(16:0/18:1)	0.839	68	96	1.370
Glycerophospholipids	PC(20:0/20:4)	0.839	88	71	−0.571
Glycerophospholipids	LPC(22:0)	0.838	88	76	−0.722
Glycerophospholipids	PC(P-18:0/20:4)	0.835	68	91	−0.631
Sterols	ChoE(16:0)	0.835	92	74	−0.519
Glycerophospholipids	PC(P-16:0/18:2)	0.826	72	76	−0.580
Sphingolipids	SM(d18:1/12:0)	0.824	80	79	−0.735
Glycerophospholipids	PC(O-16:0/20:4)	0.822	64	97	−0.587
Glycerophospholipids	PC(O-16:0/0:0)	0.821	72	85	−0.545
Glycerophospholipids	PE(P-18:0/0:0)	0.820	100	50	−0.623
Glycerophospholipids	PC(18:2/20:4)	0.816	88	68	−0.769
Glycerophospholipids	PC(18:0/20:4)	0.814	88	68	−0.537
Bile acids	Taurodeoxycholic acid	0.813	71	92	4.425
Glycerophospholipids	PC(19:0/0:0)	0.812	80	74	−0.561
Glycerophospholipids	PC(O-20:2/0:0)	0.812	88	71	−0.821

Glycerophospholipids	PE(P-18:2/0:0)	0.812	92	65	−0.859
AA derivative	D(-)-2-Aminobutyric acid	0.809	100	59	−0.499
Glycerophospholipids	PE(18:1e/22:6)	0.807	96	53	−0.558
Amino acids	Tryptophan	0.805	92	71	−0.297
Glycerophospholipids	PC(P-16:0/0:0)	0.805	64	88	−0.478
Glycerophospholipids	PE(P-18:0/18:2)	0.802	84	71	−0.784
Glycerophospholipids	PC(14:0/20:4)	0.802	84	68	−1.013

AUC, area under the receiver operating characteristic curve; FC, fold change. Colors from green to red correspond to drop or elevation of metabolite levels.

.

Table S1C. Diagnostic capacity of the metabolites in the comparison PDAC vs. Control considering the whole cohort.

Class	Metabolite	AUC	Sensitivity	Specificity	log_2FC
Amino acids	Glutamic Acid	0.937	92	88	1.570
Amino acids	Aspartic Acid	0.937	79	96	1.473
Glycerophospholipids	PE(16:0/18:1)	0.919	82	88	2.295
Sphingolipids	SM(d18:2/22:0)	0.919	92	89	−0.810
Sphingolipids	SM(39:1)	0.915	88	82	−0.907
Sphingolipids	SM(d18:2/23:0)	0.911	92	87	−1.059
Sterols	ChoE(18:3)	0.907	76	92	−1.178
Fatty esters	AC(8:0)	0.903	80	92	−1.312
Sphingolipids	SM(38:1)	0.899	96	76	−0.603
Glycerophospholipids	PE(16:0/0:0)	0.897	79	92	0.601
Sphingolipids	SM(d18:1/23:0)	0.896	96	71	−0.819
Glycerolipids	TG(58:3)	0.883	84	84	2.283
Glycerophospholipids	PE(18:0/0:0)	0.883	89	84	0.525
Glycerolipids	DG(34:0)	0.882	80	87	−1.522
Fatty acids	24:1n-9	0.879	84	88	0.959
Glycerolipids	DG(34:1)	0.876	76	88	1.056
Bile acids	TCDCA	0.876	79	88	6.369
Glycerophospholipids	PE(0:0/18:0)	0.874	76	92	0.487
Sphingolipids	SM(43:1)	0.874	100	61	−1.036
Sphingolipids	Cer(43:1)	0.872	76	87	−1.072
Sphingolipids	SM(d18:1/22:0)	0.868	88	79	−0.582
22:5n-6/22:4n-6	22:5n-6/22:4n-6	0.868	72	87	−0.604
Peptides	Gly-Gly	0.866	76	96	1.840
Bile acids	TCA	0.866	66	100	8.459
Glycerolipids	TG(58:4)	0.864	82	88	2.363
Fatty acids	20:0	0.862	68	96	0.555
Glycerolipids	TG(56:2)	0.859	84	84	1.867
Bile acids	GCA	0.857	76	92	4.903
Sphingolipids	SM(d18:2/20:0)	0.855	76	87	−0.517
Glycerolipids	TG(58:5)	0.853	89	68	1.616
Glycerophospholipids	PE(16:0/22:6)	0.851	89	68	1.272
Glycerolipids	DG(34:2)	0.849	82	80	1.094
Glycerolipids	DG(36:1)	0.848	79	76	1.231
Glycerophospholipids	PE(16:0/20:4)	0.848	87	72	1.144
Sterols	DHEAS	0.841	100	53	−1.205
Glycerolipids	TG(54:1)	0.840	79	80	1.895
Glycerolipids	TG(54:2)	0.839	71	88	1.215
Glycerolipids	TG(60:3)	0.839	84	84	1.867
Bile acids	GCDCA	0.837	63	96	2.745
Glycerophospholipids	PC(O-18:0/18:2)	0.837	100	61	−1.049
Glycerophospholipids	PC(O-16:0/18:2)	0.836	72	84	−0.771
Glycerolipids	TG(55:2)	0.836	71	88	1.445
Glycerophospholipids	PC(40:8)	0.836	80	79	−0.748
Glycerolipids	TG(50:1)	0.833	55	96	1.124
Glycerolipids	TG(50:2)	0.829	63	96	1.024
Glycerophospholipids	LPI(22:6)	0.829	58	96	1.300
Glycerolipids	TG(51:1)	0.826	74	84	1.478
Glycerolipids	TG(58:2)	0.826	76	88	2.060
Glycerophospholipids	PE(0:0/16:0)	0.826	74	84	0.464
Glycerophospholipids	PE(0:0/22:6)	0.825	66	92	0.509
Glycerolipids	TG(52:1)	0.824	79	76	1.340
Glycerolipids	TG(56:3)	0.821	68	88	1.280
Glycerophospholipids	PE(16:0/18:2)	0.821	71	80	1.393
Sphingolipids	SM(42:1)	0.816	92	71	−0.592

Glycerolipids	TG(51:2)	0.815	71	88	1.218
Glycerolipids	TG(49:1)	0.813	66	88	1.348
Fatty esters	AC(12:0)	0.812	92	61	−0.829
Glycerolipids	TG(53:1)	0.812	68	80	1.551
Glycerolipids	TG(55:3)	0.808	71	88	1.222
Sphingolipids	SM(d18:1/25:0)	0.807	68	79	−0.603
Glycerolipids	TG(53:2)	0.807	61	92	1.255
Glycerolipids	DG(32:1)	0.806	55	96	1.087
Glycerophospholipids	PC(18:2/20:4)	0.805	88	76	−0.468
Glycerolipids	TG(49:0)	0.804	84	68	1.737
Glycerophospholipids	PE(18:0/20:4)	0.804	97	52	1.107
Glycerolipids	Triacylglycerols	0.803	63	84	1.048
Glycerophospholipids	PC(O-16:0/20:3)	0.802	96	68	−0.676
Glycerophospholipids	PE(O-16:0/0:0)	0.802	92	58	−0.895
Sphingolipids	SM(d18:2/14:0)	0.802	68	89	−0.529
Glycerolipids	TG(52:4)	0.800	95	61	1.632
Glycerolipids	TG(55:4)	0.800	76	76	1.038
Glycerophospholipids	PC(20:3/20:4)	0.800	88	71	−0.749

AUC, area under the receiver operating characteristic curve; FC, fold change. Colors from green to red correspond to drop or elevation of metabolite levels.

Table S1D. Diagnostic capacity of the metabolites in the comparison dCCA vs. BPD considering the whole cohort.

Class	Metbolite	AUC	Sensitivity	Specificity	log_2FC
Sphingolipids	SM(d18:1/23:1)	0.858	79	81	0.839
Bile acids	Glycocholic acid	0.834	94	62	2.579
Bile acids	Taurocholic acid	0.823	73	83	4.096
Glycerophospholipids	PC(16:0/16:0)	0.811	76	79	0.753
Glycerophospholipids	PC(31:0)	0.805	71	81	1.233
Glycerolipids	TG(54:7)	0.800	60	91	−3.358
Fatty acids	18:3n-3	0.790	69	82	−1.368
Sphingolipids	CMH(d18:1/16:0)	0.788	82	71	0.720
Amino acids	Phenylalanine	0.785	56	90	0.482
Glycerolipids	TG(54:6)	0.783	51	100	−2.154
Glycerophospholipids	PC(O-34:1)	0.777	65	95	0.725
Amino acids	Aspartic Acid	0.768	79	69	0.836
Bile acids	GCDCA	0.766	74	71	1.571
Sphingolipids	SM(43:2)	0.760	76	69	0.872
Bile acids	TCDCA	0.760	65	81	3.496
Amino acids	Phenylalanine	0.759	71	74	0.336
Glycerophospholipids	PC(O-16:0/16:0)	0.757	62	88	0.697
Glycerophospholipids	PE(O-16:0/0:0)	0.756	93	47	−0.736
Glycerophospholipids	PC(O-22:1/20:4)	0.754	62	79	0.505
Sphingolipids	Cer(d18:1/25:0)	0.750	74	74	−0.622
Glycerophospholipids	PC(O-38:5)	0.749	47	100	0.338
Sphingolipids	SM(d18:0/15:0)	0.739	56	93	0.769
Amino Acids Derivatives	Kynurenine	0.739	85	60	0.508
Glycerophospholipids	PC(33:1)	0.737	68	81	0.820
Glycerophospholipids	PC(O-18:1/18:1)	0.733	62	79	0.655
Oxidized fatty acids	9,10-DiHOME	0.733	79	71	−0.523
Glycerophospholipids	PC(20:3/20:4)	0.730	81	59	−0.740
Bile acids	TDCA	0.727	52	95	3.074
Glycerophospholipids	PC(P-16:0/16:0)	0.725	65	88	0.569
Oxidized fatty acids	12,13-DiHOME	0.725	81	65	−1.162
Sphingolipids	Cer(d18:1/24:0)	0.723	74	71	−0.577
Sphingolipids	SM(d18:1/17:0)	0.723	88	55	0.322
Sphingolipids	SM(42:3)	0.721	35	98	0.346
Glycerolipids	TG(58:4)	0.717	50	94	−1.660
Glycerolipids	TG(58:1)	0.714	90	47	−0.629
Glycerolipids	TG(58:2)	0.714	55	79	−0.932
Fatty acids	18:0	0.714	76	59	−0.302
Bile acids	TUDCA + THDCA	0.711	50	93	3.051
Sphingolipids	Cer(d18:1/23:0)	0.709	79	68	−0.438
Sphingolipids	SM(d18:1/16:0)	0.709	85	60	0.227
Glycerolipids	TG(60:2)	0.709	64	71	−0.672

Oxidized fatty acids	x-HODE	0.709	76	65	−0.423
Glycerolipids	TG(56:1)	0.707	95	41	−0.543
Amino acids	Tyrosine	0.707	65	76	0.313
Sphingolipids	SM(d18:0/18:0)	0.705	71	71	0.836
Sphingolipids	SM(d16:1/24:1)	0.705	38	98	0.420
Glycerophospholipids	PC(O-24:1/20:4)	0.704	68	71	0.360
Fatty acids	18:2n-6	0.702	69	71	−0.433
Glycerophospholipids	PE(20:4/0:0)	0.702	81	59	−0.232

AUC, area under the receiver operating characteristic curve; FC, fold change. Colors from green to red correspond to drop or elevation of metabolite levels.

Table S1E. Diagnostic capacity of the metabolites in the comparison PDAC vs. BPD considering the whole cohort.

Class	Metbolite	AUC	Sensitivity	Specificity	log_2FC
Glycerophospholipids	PC(O-34:1)	0.814	66	90	0.951
Sphingolipids	SM(d18:1/23:1)	0.813	79	79	0.898
Glycerophospholipids	PC(P-16:0/16:0)	0.795	74	74	0.730
Glycerophospholipids	PC(16:0/16:0)	0.794	66	86	0.950
Glycerophospholipids	PC(31:0)	0.794	68	81	1.376
Glycerophospholipids	PC(O-16:0/16:0)	0.782	68	81	0.941
Glycerophospholipids	PC(O-38:5)	0.782	58	93	0.446
Glycerophospholipids	PC(O-18:1/18:1)	0.776	63	81	0.760
Glycerophospholipids	PC(O-22:1/20:4)	0.768	74	71	0.677
Sphingolipids	SM(d18:0/15:0)	0.766	55	93	1.058
Sphingolipids	CMH(d18:1/16:0)	0.765	47	98	1.030
Bile acids	Glycocholic acid	0.763	76	69	2.721
Glycerophospholipids	PC(P-16:0/18:1)	0.762	84	60	0.524
Glycerophospholipids	LPI(22:6)	0.760	55	83	1.047
Bile acids	Taurocholic acid	0.759	74	67	4.548
Sphingolipids	SM(42:3)	0.751	53	86	0.460
Glycerophospholipids	PC(O-16:0/14:0)	0.749	58	83	0.683
Glycerophospholipids	PE(38:5)	0.748	63	83	1.073
Glycerolipids	DG(38:5)	0.746	47	95	0.718
Glycerophospholipids	PC(16:0/19:1)	0.745	71	76	1.010
Glycerolipids	TG(56:6)	0.742	55	83	0.811
Sphingolipids	Cer(d18:1/16:0)	0.741	68	76	0.844
Glycerolipids	TG(54:5)	0.741	55	88	0.982
Glycerophospholipids	PC(O-40:5)	0.739	76	64	0.398
Glycerophospholipids	PC(P-36:2)	0.737	79	64	0.451
Sphingolipids	SM(d18:1/16:0)	0.734	82	60	0.352
Bile acids	TCDCA	0.734	71	69	3.763
Glycerophospholipids	PC(O-24:1/20:4)	0.733	71	76	0.518
Glycerolipids	TG(54:6)	0.733	58	86	0.942
Amino acids	Aspartic Acid	0.732	66	76	0.626
Glycerophospholipids	PI(18:0/22:6)	0.732	55	88	1.145
Sphingolipids	SM(d18:0/16:0)	0.731	50	90	0.798
Glycerophospholipids	PC(O-18:1/22:4)	0.728	66	74	0.413
Glycerophospholipids	PC(17:0/20:4)	0.727	63	76	0.779
Glycerophospholipids	PC(O-16:0/22:4)	0.725	74	64	0.475
Sphingolipids	SM(d16:1/24:1)	0.724	39	98	0.510
Glycerophospholipids	PC(16:0/18:0)	0.722	58	83	0.401
Glycerophospholipids	PC(16:0/17:0)	0.722	55	86	0.913
Glycerophospholipids	PC(18:1/22:6)	0.721	68	71	0.632
Sphingolipids	Cer(42:3)	0.719	55	81	1.177
Glycerophospholipids	PC(O-34:0)	0.719	50	95	0.812
Glycerophospholipids	PC(33:1)	0.718	63	83	1.219
Glycerophospholipids	PE(18:1/18:2)	0.717	68	81	0.751
Bile acids	GCDCA	0.712	61	74	1.566
Sphingolipids	SM(d18:1/17:0)	0.711	74	64	0.371
Glycerophospholipids	LPC(22:1)	0.711	87	52	0.374
Glycerophospholipids	PC(0:0/20:1)	0.709	89	45	0.326
Glycerophospholipids	PC(P-16:0/14:0)	0.708	45	90	0.630
Glycerophospholipids	PE(16:0/20:4)	0.707	87	48	0.732
Glycerophospholipids	PE(16:0/22:6)	0.707	84	48	0.789
Glycerophospholipids	PI(18:0/20:4)	0.707	82	55	0.915
Sphingolipids	SM(33:1)	0.707	79	57	0.432
Glycerophospholipids	PC(17:0/0:0)	0.705	76	62	0.325
Glycerolipids	TG(58:6)	0.703	34	98	0.966

Glycerophospholipids	PC(O-20:1/0:0)	0.703	68	71	0.270
Glycerolipids	TG(53:3)	0.702	61	76	0.645
Glycerophospholipids	PI(18:0/18:1)	0.701	45	93	0.591
Glycerophospholipids	PC(20:1/0:0)	0.701	79	57	0.330
Fatty acids	18:3n-3	0.698	69	74	−0.737
Sphingolipids	SM(43:2)	0.697	63	71	0.741

AUC, area under the receiver operating characteristic curve; FC, fold change. Colors from green to red correspond to drop or elevation of metabolite levels.

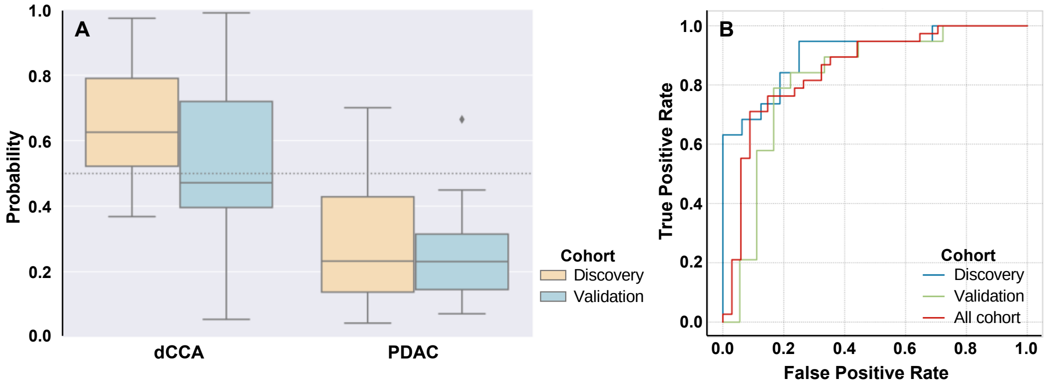

C

Cohort	AUC	Sensitivity (%)	Specificity (%)	PPV (%)	NPV (%)
**Discovery**	0.908	75.0	84.2	80.0	80.0
**Validation**	0.822	38.9	94.7	87.5	62.1
**All Cohort**	0.854	55.9	89.5	82.6	69.4

D

Class	Metabolite
AC	AC(16:0)
Cer	Cer(d18:1/24:0)
PC	PC(20:0/20:4)
PC	PC(O-16:0/20:3)
LPC	PC(20:0/0:0)
LPC	PC(0:0/20:0)
LPE	PE(P-18:2/0:0)
SM	SM(d18:2/22:0)
SM	SM(d18:2/23:0)

Figure S1. Diagnostic prediction capacity of the logistic model in dCCA vs. PDAC. (**A**) Box plot diagrams showing the probability to detect each type of tumor. (**B**) Area under the receiver operating characteristic curve (AUC) in discovery and validation cohorts and considering the whole cohort. (**C**) AUC, sensitivity, specificity, positive predictive value (PPV) and negative predictive value (NPV) of the algorithm to differentiate dCCA vs. PDAC in each cohort. (**D**) Selected metabolites included in the model. AC, acylcarnitine; Cer, ceramide; LPC, lysophosphatidylcholines; LPE, lysophosphatidylethanolamines; PC, phosphatidylcholines; SM, sphingomyelins.

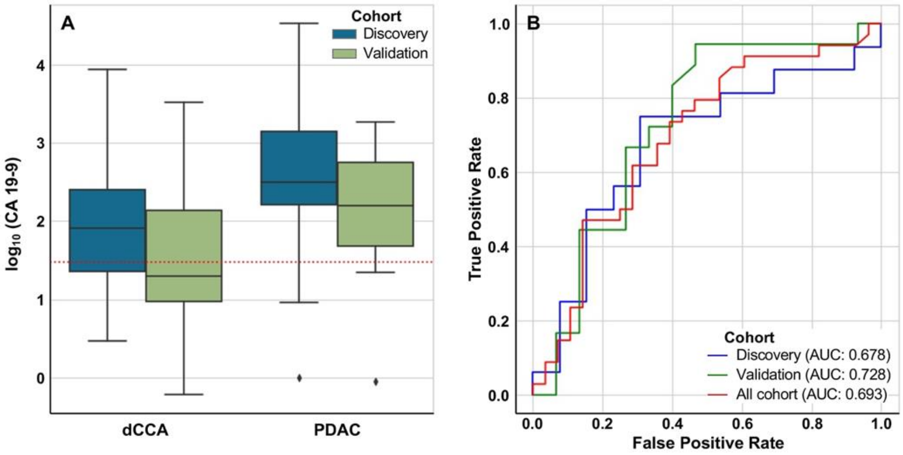

Figure S2. Diagnostic prediction capacity of CA 19-9 in dCCA vs. PDAC. (**A**) Box plot diagrams show the $\log_{10}$ CA 19-9 (cut-off of 37 IU/mL). (**B**) Area under the receiver operating characteristic curve (AUC) in discovery and validation cohorts and considering all cohorts.

© 2020 by the authors. Licensee MDPI, Basel, Switzerland. This article is an open access article distributed under the terms and conditions of the Creative Commons Attribution (CC BY) license (http://creativecommons.org/licenses/by/4.0/).
